# Evaluation of an automated blood culture detection system for sterility testing of cellular and gene therapy products

**DOI:** 10.1128/spectrum.02902-25

**Published:** 2026-03-20

**Authors:** Michael Snyder, Ashley Lawrence, Jane Reese, David Wald, Neil Anderson, Eric Ransom

**Affiliations:** 1 Department of Pathology, University Hospitals Cleveland Health System, Cleveland, Ohio, USA; 2 Cellular Therapy Operations and Quality, National Center for Regenerative Medicine, School of Medicine, Case Western Reserve University, Cleveland, Ohio, USA; 3 Department of Pathology, Case Western Reserve University School of Medicine 12304, Cleveland, Ohio, USA; 4 Department of Pathology, Louis Stokes Cleveland Veteran Affairs (VA) Medical Center 20083, Cleveland, Ohio, USA; Ascension St John Hospital, Detroit, Michigan, USA

**Keywords:** sterility testing, cellular therapy, gene therapy, automated blood culture detection, automated growth detection, bioMérieux, Virtuo, BacT/Alert, FA Plus, FN Plus

## Abstract

Sterility testing of cellular and gene therapy products is required to ensure safe infusion for the recipient. Sterility testing is often performed using broth-based media per the biopharmaceutical compendial USP <71> method. Automated blood culture systems have emerged as a potential alternative method, but data remain limited. It also remains unclear whether different cellular and gene therapy product formulations inhibit microbial growth during sterility testing, which could lead to false-negative sterility results. This study evaluated the ability to perform sterility testing using the BacT/Alert Virtuo automated blood culture system, BacT/Alert FA plus (aerobic) and FN plus (anaerobic) blood culture bottles, and two product formulations: (i) Plasmalyte-A + 2.5% human serum albumin (HSA) and (ii) Plasmalyte-A + 5% HSA + 5% dimethyl sulfoxide (DMSO). Contrived specimens were created using either formulation or a saline-only growth control inoculated with *Staphylococcus aureus*, *Bacillus subtilis*, *Pseudomonas aeruginosa*, *Clostridium sporogenes*, *Candida albicans*, or *Aspergillus brasiliensis*. Contrived specimens were prepared in blood culture sets consisting of an aerobic bottle and an anaerobic bottle and incubated for a maximum of 14 days. Bottles flagging positive underwent culture-dependent microbial identification using matrix-assisted laser desorption/ionization time-of-flight mass spectrometry (MALDI-TOF MS) for bacteria and yeast or using macroscopic and microscopic identification for filamentous fungi. The automated blood culture system method correctly flagged all contrived blood culture sets within 72 h, demonstrating no inhibitory effect on microbial recovery from either formulation. Note, both aerobic and anaerobic bottles were necessary to detect microbes with different growth preferences (e.g., strict aerobes and obligate anaerobes). Another important finding was that the addition of calcofluor staining and a Sabouraud dextrose agar (SDA) plate improved early mold detection when no organisms were detected by the initial Gram stain from a positive blood culture. Taken together, our findings suggest that two standard cellular and gene therapy product formulations do not interfere with sterility testing in an automated blood culture detection system.

**IMPORTANCE:**

Cellular and gene therapy products have emerged in recent years as an important treatment modality at large academic medical centers; however, these products must be tested for microbial contamination to ensure product safety. Compared to the gold standard, agar-based solid media method, automated blood culture systems have emerged as a potential alternative method of sterility testing. Here, we evaluated the ability of an automated blood culture detection system (BacT/Alert Virtuo) to reliably detect diverse microorganisms, ranging from bacteria to mold, in two standard cellular and gene therapy product formulations. Our results support current evidence that the use of automated blood culture detection systems is a reliable alternative method of sterility testing for different formulations of cellular and gene therapy products.

## INTRODUCTION

Cellular and gene therapy products have emerged in recent years as an important treatment modality at large academic medical centers. The Food and Drug Administration (FDA) has approved over 30 cellular and gene therapy products, such as chimeric antigen receptor T-cell (CAR-T) therapy for the treatment of diffuse large B-cell lymphoma and other hematologic malignancies (1). Approval of these treatments is expected to accelerate in the coming years as they are used to treat a wider range of diseases, such as autoimmune diseases and solid tumors (2). Notably, these biopharmaceutical products must be tested for microbial contamination to ensure product safety prior to infusion in the recipient (3). The compendial USP <71> sterility testing method is the biopharmaceutical gold standard for testing stem cell transplant products and is the basis for testing more sophisticated cellular therapy products (4–6). Using this method, products are directly inoculated into two broth media (tryptic soy broth and thioglycolate), incubated for 14 days, and periodically evaluated for signs of visible macroscopic growth.

There is growing evidence that automated growth-based detection systems are a potential alternative to the compendial USP <71> (6–10). Automated blood culture systems have been used for ensuring the purity of hematopoietic stem cells for stem cell transplants, as well as recovery of microorganisms from non-blood sterile body fluids (11). However, their reliability in the context of modern cell therapy remains under investigation. Although not FDA-approved for this purpose, automated blood culture systems offer potential benefits. They provide automated continuous growth detection and require minimal technologist handling, thereby minimizing the possibility of contamination and freeing laboratory personnel for other tasks. Automated blood culture systems are also readily available at most clinical laboratories, offering a more rapid turnaround time and negating the need for costly send-out testing.

Importantly, cellular and gene therapy products are suspended, stored, and/or infused in special cryoprotective solutions to ensure cellular viability and functionality following infusion into the recipient (12). It has been theorized that these formulations could impact microbial recovery in sterility testing, leading to false-negative sterility results. Because these formulations are rapidly evolving, it is important for laboratories to perform ongoing evaluations of the formulations to ensure sterility testing results are accurate.

Here, we evaluated the ability of an automated blood culture detection system to reliably detect diverse microorganisms, ranging from bacteria to mold, in two standard cellular and gene therapy product formulations: Plasmalyte-A + 2.5% human serum albumin (HSA) and Plasmalyte-A + 5% HSA + 5% dimethyl sulfoxide (DMSO). We used the BacT/Alert Virtuo (bioMérieux) continuous monitoring blood culture system, along with aerobic BacT/Alert FA Plus and anaerobic FN Plus bottles. In addition, this study included an 8-year retrospective review of cellular therapy sterility testing to better understand positivity rates and likely contaminant microorganisms.

## MATERIALS AND METHODS

### Selection of materials and sample organisms

The BacT/Alert Virtuo continuous monitoring blood culture system was used to detect the growth of six representative organisms in two standard cellular therapy formulations. The six organisms were *Staphylococcus aureus, Bacillus subtilis, Pseudomonas aeruginosa, Clostridium sporogenes, Candida albicans,* and *Aspergillus brasiliensis*. These organisms are specified in the compendial USP <71> method for sterility testing and represent a diverse set of microorganisms, ranging from bacteria to mold, including both aerobes and anaerobes.

The two cell therapy formulations evaluated were Plasmalyte-A + 2.5% HSA and Plasmalyte-A + 5% HSA + 5% DMSO. These formulations were selected in collaboration with our institution’s Cell Therapy Center because these standard solutions enable cryopreservation of cells for therapeutic applications, such as CAR-T therapy, ensuring the viability and functionality of human cell and tissue products following infusion into the recipient. A growth control formulation (saline) was also used to confirm microbial viability since saline should not impact microbial growth. All conditions under investigation had two replicates tested in parallel to evaluate reproducibility.

### Methodology and data collection

**Fig 1:**
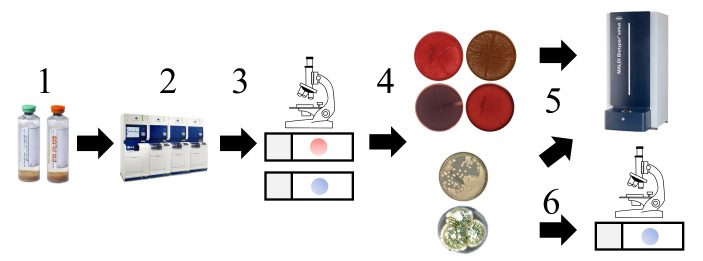
(1) Contrived samples with different cellular therapy product formulations and organisms were inoculated into aerobic and anaerobic bottles. (2) Bottles were incubated in the BacT/Alert Virtuo automated blood culture detection system. (3) Bottles flagging positive underwent Gram staining and, if negative, calcofluor staining. (4) Bottles were always subcultured to solid agar media for bacterial (top) and fungal (bottom). (5) Bacteria and yeast were identified by MALDI-TOF MS. (6) Filamentous fungi were identified by macroscopic and microscopic observation.

Per the manufacturer’s guidelines, bioMérieux Bioballs containing the six lyophilized organisms were reconstituted by pipetting rehydration solution to achieve 50 colony-forming units (CFUs) per 100 µL. Total resuspension volumes ranged from 1,056 to 1,146 µL. Colony counts were performed by plating 100 µL of the resuspension (~50 CFUs) to tryptic soy agar plates, which confirmed all inoculations were between 10 and 100 CFUs. The remaining suspensions of organisms were diluted 1:10 into the two cell therapy formulations and a saline growth control. Bottles were prepared in sets/pairs (aerobic BacT/Alert FA Plus and anaerobic FN Plus), inoculated with 50 CFUs per bottle, and incubated on the BacT/Alert system for a maximum of 14 days. A total of 18 contrived culture sets/pairs were tested with two replicates, except for the saline-only control. The subsequent workflow is shown in Fig. 1. Negative bottles (i.e., bottles that never flagged positive) were not stained and not subcultured at the end of the incubation. Positive bottles underwent primary staining (Gram stain and, if negative, calcofluor white) and were always subcultured to solid agar media (blood, MacConkey, chocolate, CDC anaerobic, and Sabouraud dextrose agar [SDA]). Samples were submitted for standard-of-care microbial identification using matrix-assisted laser desorption/ionization time-of-flight mass spectrometry (MALDI-TOF MS) for bacteria and yeast or using macroscopic and microscopic identification for the filamentous fungus. Bottles that flagged positive on the Virtuo blood culture system and no organisms were seen on the Gram stain were (i) reloaded on the Virtuo system, (ii) plated to agars (blind subculturing), and (iii) stained with calcofluor fluorescent stain.

### Retrospective review

All sterility testing for cellular and gene therapy products from 1 January 2016 to 30 June 2025 was evaluated for positivity and microorganisms recovered. This historic sterility testing followed a similar workflow as described above, except from 2016 to 2017 the BD BACTEC FX system was used, from 2017 to 2024 the Virtuo system with SA aerobic and SN anaerobic BacT/Alert bottles was used, from 2016 to 2025 the calcofluor stain was not used unless a fungal mass (i.e., fungal ball) was observed in the blood culture bottle, and from 2016 to 2025 a SDA plate was added only if fungal elements were observed macroscopically or microscopically. Testing for research studies, validations, proficiency, and other quality assurance projects was excluded from analyses.

### Statistical analysis

Analyses were performed using two-tailed unpaired *t*-tests in Microsoft Excel to determine a statistically significant difference in time to detection between microbes reconstituted in saline-only growth control versus microbes reconstituted in cell therapy formulations.

## RESULTS

### General performance of sterility testing

To evaluate if standard cell therapy formulations impact microbial recovery during sterility testing, contrived blood culture sets were prepared and tested using the BacT/Alert Virtuo automated blood culture system with aerobic FA Plus and anaerobic FN Plus bottles. The BacT/Alert Virtuo automated blood culture system correctly flagged all contrived culture sets/pairs (18/18) within 72 h (see Table 1). Of note, two anaerobic bottles of *C. albicans* did not meet the USP <71> standards of suitability as defined by detection within 5 days of incubation in the presence of product; however, the corresponding aerobic bottles in the set did meet the standards. The overall median time to positivity per bottle of experimental samples was 15 h 48 min, with a maximum time to detection of 12 days 12 h in a single anaerobic bottle of *C. albicans*. Because the time to positivity of the second positive bottle in a set is less impactful, analyses for time to positivity were performed for only the first positive bottle. The median time to positivity for the first positive bottle was 13 h 21 min, with a maximum time to detection of 45 h 3 min in a set of *A. brasiliensis*.

**TABLE 1:** Results of 18 contrived culture sets with time-to-detection metrics^*a*^

Organism	Cell therapy formulation	Bottle type	Culture growth	Time to detection (h)	Initial stain	Identification	Results matched expected?
*S. aureus*	P-A + 2.5% HSA	A	2 of 2	12.1; 15.4	×2 GPCCL	×2 *S. aureus*	Yes (2 of 2)
		An	2 of 2	16.3; 12.8	×2 GPCCL	×2 *S. aureus*	Yes (2 of 2)
	P-A + 5% HSA + 5% DMSO	A	2 of 2	13.1; 13.1	×2 GPCCL	×2 *S. aureus*	Yes (2 of 2)
		An	2 of 2	15.8; 15.8	×2 GPCCL	×2 *S. aureus*	Yes (2 of 2)
	Saline (control)	A	1 of 1	12.9	×1 GPCCL	×1 *S. aureus*	Yes (1 of 1)
		An	1 of 1	17.6	×1 GPCCL	×1 *S. aureus*	Yes (1 of 1)
*B. subtilis*	P-A + 2.5% HSA	A	2 of 2	10.2; 9.9	×2 GPB	×2 *B. subtilis*	Yes (2 of 2)
		An	1 of 2	19.4	×1 GPB	×1 *B. subtilis*	Yes (1 of 2)
	P-A + 5% HSA + 5% DMSO	A	2 of 2	10.2; 10.0	×2 GPB	×2 *B. subtilis*	Yes (2 of 2)
		An	2 of 2	26.7; 22.3	×2 GPB	×2 *B. subtilis*	Yes (2 of 2)
	Saline (control)	A	1 of 1	11.2	×1 GPB	×1 *B. subtilis*	Yes (1 of 1)
		An	1 of 1	24.7	×1 GPB	×1 *B. subtilis*	Yes (1 of 1)
*P. aeruginosa*	P-A + 2.5% HSA	A	2 of 2	13.3; 12.9	×2 GNB	×2 *P. aeruginosa*	Yes (2 of 2)
		An	1 of 2	58.8	×1 GNB	×1 *P. aeruginosa*	Yes (1 of 2)
	P-A + 5% HSA + 5% DMSO	A	2 of 2	12.9; 13.0	×2 GNB	×2 *P. aeruginosa*	Yes (2 of 2)
		An	2 of 2	78.7; 59.3	×2 GNB	×2 *P. aeruginosa*	Yes (2 of 2)
	Saline (control)	A	1 of 1	13.7	×1 GNB	×1 *P. aeruginosa*	Yes (1 of 1)
		An	1 of 1	74.8	×1 GNB	×1 *P. aeruginosa*	Yes (1 of 1)
*C. sporogenes*	P-A + 2.5% HSA	A	0 of 2	NA	NA	NA	Yes (2 of 2)
		An	2 of 2	13.4; 14.4	×2 GPB	×2 *C. sporogenes*	Yes (2 of 2)
	P-A + 5% HSA + 5% DMSO	A	0 of 2	NA	NA	NA	Yes (2 of 2)
		An	2 of 2	14.4; 15.4	×2 GPB	×2 *C. sporogenes*	Yes (2 of 2)
	Saline (control)	A	1 of 1	27.1	×1 GNB	×1 *Ralstonia* spp.	Yes, w/contam
		An	1 of 1	14.6	×1 GPB	×1 *C. sporogenes*	Yes (1 of 1)
*C. albicans*	P-A + 2.5% HSA	A	2 of 2	25.9; 28.2	×2 yeast	×2 *C. albicans*	Yes (2 of 2)
		An	1 of 2	299.7	×1 yeast	×1 *C. albicans*	Yes (1 of 2)
	P-A + 5% HSA + 5% DMSO	A	2 of 2	29.2; 26.5	×2 yeast	×2 *C. albicans*	Yes (2 of 2)
		An	1 of 2	176.9	×1 yeast	×1 *C. albicans*	Yes (1 of 2)
	Saline (control)	A	1 of 1	31.0	×1 yeast and GNB	×1 *C. albicans* and *Ralstonia* spp.	Yes, w/contam
		An	0 of 1	NA	NA	NA	Yes (1 of 1)
*A. brasiliensis*	P-A + 2.5% HSA	A	2 of 2	42.3; 42.3	×2 septate hyphae	×2 *A. brasiliensis*	Yes (2 of 2)
		An	0 of 2	NA	NA	NA	Yes (2 of 2)
	P-A + 5% HSA + 5% DMSO	A	2 of 2	45.1; 40.7	×2 septate hyphae	×2 *A. brasiliensis*	Yes (2 of 2)
		An	0 of 2	NA	NA	NA	Yes (2 of 2)
	Saline (control)	A	1 of 1	28.3	×1 GNB	×1 *Ralstonia* spp.	No, w/contam (0 of 1)
		An	0 of 1	NA	NA	NA	Yes (1 of 1)

^*a*^ The automated detection system correctly detected all contrived culture sets (18/18) within 72 h*. Ralstonia* spp. were unexpectedly co-detected in one set of *C. sporogenes *and* C. albicans*, and *Ralstonia* was detected instead of *A. brasiliensis *in the saline-only growth control. Repeat sterility testing using fresh, sterile saline resolved all previous discrepancies. Bottles inoculated with mold correctly flagged positive on the BacT/Alert Virtuo, but staining and agar growth remained negative (except for one sample contaminated with *Bacillus* spp.). These no-organism-seen bottles were reloaded on the Virtuo and flagged positive again <24 h, from which septate hyphae were observed by calcofluor staining and mold was recovered and identified. P-A, Plasmalyte-A; A, aerobic bottle; An, anaerobic bottle; GPCCL, gram-positive cocci in clusters; GPB, gram-positive bacilli; GNB, gram-negative bacilli; NA, testing was not performed because the blood culture was negative.

To establish baseline time to positivity without potential inhibitory substances, the median time to positivity was calculated using the sets reconstituted in saline only, which was 14 h 9 min. Sets reconstituted in Plasmalyte-A + 2.5% HSA had a median time to detection of 13 h 21 min, while sets reconstituted in Plasmalyte-A + 5% HSA + 5% DMSO had a median time to detection of 13 h and 46 min. There was no statistically significant difference in time to detection between saline only and Plasmalyte-A + 2.5% HSA (*P* = 0.83), nor saline only and Plasmalyte-A + 5% HSA + 5% DMSO (*P* = 0.77).

### Organism-specific performance of sterility testing

Median time to detection per bottle varied by organism. *S. aureus*: 14 h 17 min; *B. subtilis*: 10 h 13 min*; P. aeruginosa:* 13 h 18 min; *C. sporogenes*: 14 h 24 min; *C. albicans*: 28 h 41 min; *A. brasiliensis*: 42 h 18 min. Because the first positive bottle in a set is usually more clinically significant, analyses were also performed for only the first bottle. *S. aureus*: 12 h 57 min; *B. subtilis*: 10 h 5 min; *P. aeruginosa*: 12 h 56 min; *C. sporogenes*: 14 h 24 min; *C. albicans*: 27 h 20 min; *A. brasiliensis*: 42 h 18 min. A possible exception was for *A. brasiliensis,* where the saline growth set flagged after 28 h 15 min, while the sets in the cell therapy formulations flagged after 42 h 18 min (median time to detection).

In terms of individual bottles, the system correctly flagged positive and led to sample organism identification for 10/10 bottles of *S. aureus*, 9/10 bottles of *B. subtilis*, 9/10 bottles of *P. aeruginosa*, 5/5 anaerobic bottles (0/5 aerobic) of *C. sporogenes*, 7/10 bottles of *C. albicans*, and 3/5 aerobic bottles (0/5 anaerobic) of *A. brasiliensis*. There was one sample each of *B. subtilis* and *P. aeruginosa* which did not grow in the anaerobic bottles; however, both organisms are known to have preferential aerobic growth. *C. sporogenes* grew only in anaerobic bottles, which is to be expected for an obligate anaerobe. Three samples of *C. albicans* did not grow in the anaerobic bottles*,* although this organism is known to prefer aerobic conditions. While all five aerobic bottles of *A. brasiliensis,* a strict aerobe, flagged positive on the Virtuo, mold was not readily identified in two samples, requiring additional steps to optimize mold recovery and identification.

### Optimization of mold detection

Blood culture bottles flagging positive underwent Gram staining (Fig. 1). The initial Gram stain correctly correlated with culture findings in 46 of 46 bottles, excluding molds. A major challenge during the study was the detection of mold by the initial Gram stain. In fact, none of the bottles with *A. brasiliensis* were initially reported as positive with mold. The presence of mold was apparent due to visible macroscopic growth, with a fungal ball/clump seen in the bottle. Only after fluorescence staining with Calcofluor white were the bottles noted to contain a septate mold.

Another approach to detect mold is to look for growth on agar. At our institution, positive blood bottles are not routinely inoculated to fungal agar, unless a mold is detected by initial microscopy. The inclusion of fungal agar was found to be important, with the most reliable recovery on fungal agars (5/5) when compared to blood (2/5), chocolate (0/5), MacConkey (0/5), and CDC anaerobic agar (0/5). Given the low volume and positivity rate of cellular therapy sterility cultures, it is reasonable for laboratories to always perform calcofluor white staining and plate to fungal agar to optimize early mold detection.

### Detection of an environmental contaminant

An unexpected finding during the study was the additional recovery of *Ralstonia* in the saline-only controls of *C. sporogenes* and *C. albicans*, while *Ralstonia* was detected instead of *A. brasiliensis* in the saline-only control. *Ralstonia* was not found in any of the samples with cellular therapy formulations. Additional testing directly from the saline stock bottle found *Ralstonia* as well, confirming the saline stock was contaminated. This was an unexpected finding to all involved and showcased the ability of the proposed sterility testing approach to detect unexpected contamination. Repeat sterility testing using fresh, sterile saline resolved all previous discrepancies and successfully grew *C. sporogenes*, *C. albicans*, and *A. brasiliensis*.

### Retrospective review

To better understand the historic trends of sterility testing, an 8-year retrospective review was performed. There were 1,667 sterility tests performed in our laboratory, spanning 1,006 patients. The overall positivity rate was only 2.2%, with the average yearly positivity rate ranging from 0% to 5.2%. Over the 8 years, there were 36 positive cultures: *Cutibacterium* (*Propionibacterium*) *acnes* (*n* = 21), *Staphylococcus epidermidis* (*n* = 3), *Staphylococcus saccharolyticus* (*n* = 3), *Staphylococcus capitis* (*n* = 2), coagulase-negative staphylococcus (unspecified, *n* = 2), *Staphylococcus hominis* (*n* = 1), *Brevundimonas vesicularis* (*n* = 1), *S. aureus* (*n* = 1), gram-positive cocci in clusters (unspecified, *n* = 1), and pleomorphic gram-positive bacilli (unspecified, *n* = 1). *S. saccharolyticus* and *C. acnes* were only recovered from the anaerobic bottle, highlighting the importance of routinely using an aerobic and anaerobic bottle for sterility testing. Of note, a mold was never recovered despite the 14-day bottle incubation.

## DISCUSSION

Sterility testing of cellular and gene therapy products is required to ensure product safety prior to infusion in the recipient. Compared to the gold standard compendial USP <71> method, automated blood culture systems have emerged as a potential alternative method of sterility testing (6–9, 13–16). Although there have been several publications evaluating the use of automated blood culture systems for sterility testing of cell-based therapeutics, information remains limited on the impact of different cell therapy product formulations, especially for newer formulations. An ongoing concern is whether different cell therapy product formulations inhibit microbial growth, which could lead to false-negative sterility results (6, 7). This includes challenges related to the recovery of fungal contaminants (8). Our study focused on the potential impact of different product formulations on sterility testing using the BacT/Alert Virtuo system with FA Plus and FN Plus bottles. Our results demonstrated excellent sensitivity overall by the automated blood culture detection system for detecting diverse microorganisms in standard cell therapy formulations. The BacT/Alert system correctly flagged all contrived blood culture sets within 72 h, demonstrating no inhibitory effect on microbial recovery from either formulation.

Turnaround time is of critical importance, both for the treatment of severely ill patients and due to the short product shelf life. Automated blood culture systems are readily available at most clinical laboratories, reducing the need for slow and costly send-out testing since samples are tested in-house. Several studies have demonstrated that the BacT/Alert Virtuo system significantly decreases time to detection of clinical blood culture isolates when compared to previous methods (17–21). Furthermore, our time-to-detection results here showed no significant difference in median time to positivity between sets reconstituted in normal saline (14 h 9 min) versus Plasmalyte-A + 2.5% HSA (13 h 21 min) and Plasmalyte-A + 5% HSA + 5% DMSO (13 h 46 min). These data are also consistent with time to positivity for blood cultures used for routine patient care in the clinical laboratory (22, 23). This may be unexpected given the presence of microbial inhibitory substances, such as DMSO, in select products.

Considering that all sets flagged positive in under 72 h, it may be reasonable in the future to consider shortening the incubation period from 14 days to 4–7 days, thereby enabling more rapid product release. Khuu et al. reported both BacT/ALERT and Bactec are more sensitive, faster in time to detection, less prone to false-positive results, and less labor-intensive compared to the compendial USB <71> method (13). The authors concluded that a 7-day incubation period is sufficient to detect clinically relevant microorganisms. Additionally, Lysák et al. demonstrated positivity within 48 h, even with a low inoculum of 10 CFUs (14). Taken together, these reports are similar to our findings, which suggest an incubation period beyond 7 days is unnecessary.

In addition to faster turnaround time, automated blood culture systems provide continuous growth detection, freeing laboratory personnel for other essential tasks. This is especially important considering current national laboratory workforce shortages. Furthermore, samples are tested in a closed system and require minimal technologist handling, thereby minimizing the possibility of environmental contamination during testing.

Both aerobic and anaerobic bottles were needed to identify microbes with varied growth requirements, such as obligate anaerobes and strict aerobes. For example, the obligate anaerobe *C. sporogenes* was detected only in anaerobic BacT/Alert FN Plus blood culture bottles, and the strict aerobe *A. brasiliensis* was detected only in aerobic FA Plus blood culture bottles. From the retrospective review data, *S. saccharolyticus* and *C. acnes* were recovered only from the anaerobic bottle. This highlights the importance of routinely using both an aerobic and anaerobic bottle for sterility testing, particularly when there are global supply shortages of blood culture bottles.

Another important finding of our study was that mold recovery and identification required subculture optimization. A previous study recommended terminal visual inspection of the bottle to identify gross mold contamination that fails to be detected by automatically detected by the system (8). This approach was not necessary in our study. The addition of calcofluor white staining and SDA plate enhanced early mold detection when no organisms were identified by the initial Gram stain following blood culture bottle positivity. Our laboratory technicians were subsequently re-trained to add a calcofluor stain and an SDA plate to routine specimens with negative Gram staining and a positive blood culture bottle. Technicians were also reminded to examine blood culture bottles for signs of visible mold growth and to agitate these bottles prior to liquid dispensing to ensure adequate mold recovery. Given the infrequency of mold from routine blood cultures, it is important that laboratory staff are properly trained and reminded of key differences with sterility testing and may warrant unique workflows, such as reflexing to calcofluor white staining, if Gram stain negative, and plating to fungal agar. Importantly, FDA-approved indications for use of the FA Plus bottle include only detection of bacteria and yeast in clinical isolates, so their use for mold detection (especially as an environmental contaminant) is not claimed. In addition, *A. brasiliensis* was the only mold tested in our protocol, and future work should evaluate other molds to determine the suitability of our method broadly.

Temperature is an important consideration for sterility testing in a clinical microbiology laboratory. Our findings suggest incubation at 35°C–37°C is sufficient for the recovery of the microorganisms tested. However, the compendial USP <71> method includes tryptic soy broth at 20°C–25°C, which theoretically would benefit environmental microorganisms that prefer lower incubation conditions. In a comparative study, England et al. showed superior performance when both the aerobic and anaerobic bottles were incubated at 30°C–35°C (8). This elevated temperature is more practical for clinical laboratories since it is uncommon to have a dedicated automated blood culture system for room temperature incubation.

As previously mentioned, one of the potential benefits of automated systems is a reduced risk of contamination during testing due to minimal technologist handling of specimens. Over the last 8 years at our institution, using an automated system, sterility testing of cell therapy products had an overall positivity rate of 2.2% (a total of 36 contaminants from 1,667 sterility tests performed). The most common contaminants were commensal skin flora, such as *C. acnes* (*n* = 21) and *S. epidermidis* (*n* = 3), which may be due to poor collection practices or improper specimen handling. The epidemiological profile was similar to previous reports that listed coagulase-negative staphylococci and *Cutibacterium* species as common contaminants (10, 16). Interestingly, Cundell et al. reported *Ralstonia* species as a common contaminant. In our study, *Ralstonia* was unexpectedly detected or co-detected in multiple samples, highlighting the challenge of eliminating environmental contamination even with automated systems. *Ralstonia* is a common water-borne environmental organism and is associated with water contamination. Here, the unexpected recovery of *Ralstonia* proved to be a useful and unexpected test of the sterility testing process and underscores the importance of using fresh, sterile saline. Although FA Plus and FN Plus bottles are intended for the detection of clinical isolates and not environmental contaminants, these bottle types were sufficient to detect contamination, albeit contrived, in this study. Overall, these findings demonstrated the ability of the proposed sterility testing approach to successfully detect microorganisms without using the USP <71> method, even though automated systems and their respective bottles are optimized for the recovery of clinical isolates.

A limitation of our study was the small number of microorganisms tested at a single institution. A wider range of challenge organisms, including *C. acnes*, which was frequently detected in our retrospective review, may reveal different detection rates. Another limitation is the integration into the routine clinical laboratory workflows. Negative sterility testing can be easily integrated, but positive samples may pose challenges when coupled with other routine testing. For example, it is important that laboratories review considerations for mold detection, such as the addition of a calcofluor stain and SDA plate, when indicated. Finally, sterility testing performed by automated blood culture systems may not sufficiently detect fastidious bacteria, *Mycobacterium* species, *Mycoplasma* species, and other microorganisms requiring agar for growth (6, 24–27). Given the importance and need for sterility testing of cellular and gene therapy products, more studies are needed before widespread adoption.

In summary, our findings support the use of automated blood culture detection systems as a reliable alternative method of sterility testing for different formulations of cellular and gene therapy products. While incorporating this approach into laboratory workflows will depend on each laboratory’s available resources, staffing, and expertise, automated blood culture systems have advantages compared to the biopharmaceutical compendial USP <71> method.
